# Application of Cost Effective and Real-Time Resistivity Sensor to Study Early Age Concrete

**DOI:** 10.3390/s23177525

**Published:** 2023-08-30

**Authors:** José Roberto Tenório Filho, Yawar Abbas, Jos Oudenhoven, Stijn Matthys

**Affiliations:** 1 Magnel-Vandepitte Laboratory, Department of Structural Engineering and Building Materials, Faculty of Engineering and Architecture, Ghent University, Tech Lane Ghent Science Park, Campus A, Technologiepark Zwijnaarde 60, B-9052 Ghent, Belgium; roberto.tenorio@ugent.be; 2 IMEC at Holst Centre, 5656 AE Eindhoven, The Netherlands; yawar.abbas@imec.nl (Y.A.); jos.oudenhoven@imec.nl (J.O.)

**Keywords:** structural monitoring, sensors, concrete, resistivity, PCB

## Abstract

Concrete is a widely used construction material, demanding strict quality control to maintain its integrity. The durability and lifespan of concrete structures rely heavily, amongst other factors, on the characteristics of fresh and early age concrete, which are strongly dependent on the curing process. To ensure long-term durability, it is crucial to assess concrete properties throughout construction and verify compliance with design specifications. Currently, electrical resistivity-based sensors are available and used for quality control and monitoring, however, these sensors tend to be costly or only measure at a single location within the concrete cover. This study introduces a printed circuit board (PCB)-based array of electrodes capable of measuring concrete resistivity profiles across the concrete cover, from its fresh state to early age development. In this work, the feasibility of such resistivity PCB-sensors, novel for concrete, is evaluated under laboratory conditions. The sensors exhibit a promising performance in monitoring the efficiency of concrete curing under various conditions. Additionally, they successfully evaluate the effectiveness of internal curing (in our study, promoted by superabsorbent polymers) during the initial stages of hardening. This sensor array provides a valuable tool for monitoring the curing of concrete at early age, and showcases a preliminary solution that could be further developed to ensure long-term performance of concrete infrastructure.

## 1. Introduction

Concrete is one of the most widely used building materials in the world and is used in most infrastructure today. Therefore, it is critical to ensure that its quality is maintained during construction [1]. Concrete can be seen as an artificial rock, obtained from the mixture of natural aggregates glued together by a mineral binder (mostly cement) which upon reaction with water is hardened and develops mechanical strength. The appropriate hydration of cement is of utmost importance for the properties of the material [2,3], and it might be influenced by environmental conditions such as ambient temperature and humidity.

In this context, one very important stage in the production process of a concrete structure is the so-called curing, which aims to reduce water loss in the concrete structure at very early ages. One way of accomplishing this is by covering the fresh concrete surface to prevent any evaporation of water [4]. Alternatively, curing agents can be used to either prevent/reduce the drying by supplying additional water to the structure (external curing [5]) or by incorporating water-filled inclusions into concrete to supply curing water uniformly from the inside (internal curing [6]). Due to the dependence of the hydration process on the water availability and the realistic environmental conditions of concrete structures, evaporation is almost inevitable, thus, the durability and service life of concrete structures rely greatly on the efficiency of the curing process which might limit the cement hydration and strength development (both particularly critical to ensuring the long-term durability of concrete infrastructure) [7]. In addition, concrete structures undergoing inappropriate curing are more often related to a poor microstructure development, resulting in more permeable matrices with higher susceptibility to the movement of aggressive gases and/or liquids from the environment [8]. It is, therefore, fundamentally important to be able to properly monitor the curing process of concrete structures to assure the quality of the concrete from an early age.

In terms of monitoring methods, the electrical resistivity (ER) measurement inside concrete has been used in a few studies as a moisture and durability indicator [9,10], and some correlations between the ER and the compressive strength of concrete over time have also been investigated [11,12,13]. The ER of concrete can be measured on the surface or via embedded electrodes in concrete where an alternating current is applied between these electrodes and the potential drop is measured. The ER is mainly influenced by the (1) concrete pore size and distribution, (2) moisture content, and (3) ions present in the concrete [14]. The moisture content in early age concrete is an indicator of the efficiency of concrete curing [15,16] and the ER measurement can be used to measure it [10,17,18,19]. However, most of the ER-based sensors presented in the literature [10,20,21] or available commercially are either expensive or only measure at one location in the concrete cover depth. For reliable measurement of the curing process in concrete, an array of ER electrodes that can measure resistivity profiles over the concrete cover depth can be beneficial [22].

In this study, we present a PCB-based array of resistivity sensors that can measure concrete resistivity from the fresh state of concrete throughout the early age concrete development, and correlate the measurements with the ongoing hydration and hardening of concrete under different curing conditions, simulating real case applications. PCB-based sensors are less expensive than other sensors, as they are manufactured using standard processes that facilitate mass production [23]. Such cost-effectiveness will enable the implementation of a network of sensors throughout the structure, providing more representative values and better estimates of curing efficiency and strength development (among other properties).

## 2. Material and Methods

The experimental setup consisted of concrete cubes (side length of 150 mm) of two different mix designs embedded with the PCB-based ER sensors for a period of 7 days while curing under different conditions. In this section, we present details about the concrete mixtures used in the study, about the sensor design, and the experimental setup.

### 2.1. Concrete Mixtures and Curing Conditions

Two concrete mixtures were studied: a first mixture (Concrete A, without any curing agent) and a second mixture (Concrete B, containing superabsorbent polymers (SAPs) as an internal curing agent). 

**Figure 1:**
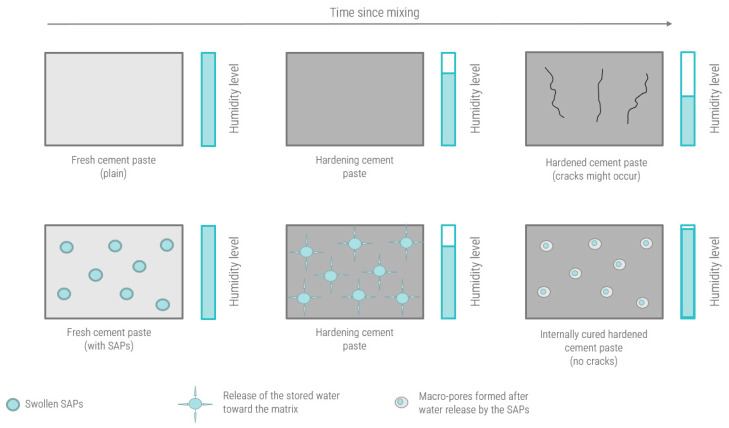
Simplified working mechanism of SAPs as internal curing agents in the hardening cement paste.

Superabsorbent polymers consist of a natural and/or synthetic water-insoluble 3D network of polymeric chains crosslinked by chemical or physical bonding [24]. They possess the ability to take up a significant amount of liquids from the environment (in amounts up to 1500 times their own weight) [25] and form an insoluble gel [26]. During concrete mixing, the polymers absorb water, and once the humidity levels inside the hardening concrete start to decrease, this amount of absorbed water can be slowly and controllably released due to drying or osmotic pressure, consequently causing the SAPs to shrink in size [27]. A simplified representation of the working mechanism of SAPs as internal curing agents in cementitious materials is depicted in Figure 1.

An overview of concrete mixtures A and B is presented in Table 1. For both mixtures, Portland cement type CEM I 52.5 N (Holcim, Antwerp, Belgium) was used, as well as river sand (aggregate size range of 0/4 mm), gravel (aggregate size ranges of 2/8 mm and 8/16 mm), and a commercial SAP (SNF Floerger, Saint-Avold, France) based on a cross-linked acrylate copolymer produced through bulk polymerization (with a mean particle size (D_50_) of 360 µm and absorption capacity of 21 g/g [28]).

In the mixing procedure, the dry materials were first mixed for 1 min (including SAPs, when present), then the mixing water was added and mixed for an additional 2 min. When SAPs were present, the additional entrained water was added during the third minute and the mixing proceeded for an additional 2 min. The total mixing time was 3 min for Concrete A and 5 min for Concrete B. 

For Concrete A, fifteen cubes (150 mm) were produced by pouring the fresh concrete in a cubical mold, and were kept under different curing conditions: (1) room 20/90, (2) room 20/60, and (3) room 32/60. The details of the ambient conditions in these rooms are given in Table 2. Three of the cubes were used for data collection with the sensors, and the remaining cubes were used for compressive strength tests performed at the ages of 1, 3, and 7 days after casting (in triplicate for each curing condition). For Concrete B, two cubes were produced for data collection with the sensors and were kept in room 20/60. The cubes were transported to the respective rooms immediately after pouring the concrete in the mold and then demolded around 24 h later. The data collection started right after storing the cubes in the respective curing locations, and measurements were performed every 12 s for a period of 7 days.

### 2.2. ER Sensor Design and Experimental Setup

**Figure 2:**
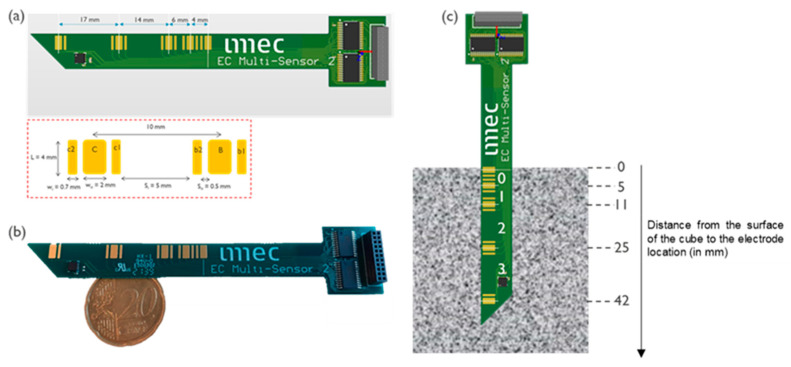
(**a**) ER sensor design based on the array of electrodes on a PCB substrate, (**b**) snapshot of the sensor, and (**c**) schematic of sensor embedded in concrete.

The array of ER electrodes used in this study allowed the monitoring of four different locations over the concrete cover depth, as shown in Figure 2. The sensor design had a sharp tip, to facilitate insertion in the viscous concrete mixture and ensure that no air-pockets were trapped at the electrode side. 

The sensor was composed of hard-plated gold electrode on a PCB substrate. The fabrication technique was in line with the standard PCB board manufacturing. 

The levels of the ER sensor in the concrete cover were denoted as level 0, 1, 2 and 3, with respect to the concrete surface. At level 0, the sensing electrodes were positioned at 1 and 5 mm from the concrete surface. Level 1 was defined as a measurement between electrodes at 5 and 11 mm from the surface, level 2 between electrodes at 11 and 25 mm, and finally, level 3 was measured between electrodes at 25 and 42 mm from the concrete surface. These distances were chosen to collect higher spatial resolution of the resistivity measurement close to the concrete surface where the change in resistivity values was higher compared with the innermost depth (50 mm) of the concrete cover depth. 

The sensor was based on impedance spectroscopy, where an alternating current (AC) excitation voltage was applied between a pair of electrodes, and the resulting current response was measured. Using Ohms law, the impedance was evaluated. The resulting impedance was a combination of real and complex parts with magnitude and phase shift of the output signal depending on the applied frequency. The impedance at which the phase shift of the output response was zero gave the resistance or resistivity of the medium between the electrodes. The details of the electrode design are given in Table 3. For this design, the applied voltage amplitude and frequency (at which the phase shift between input and output signal is zero) were found to be 50 mV and 3 kHz, respectively.

**Figure 3:**
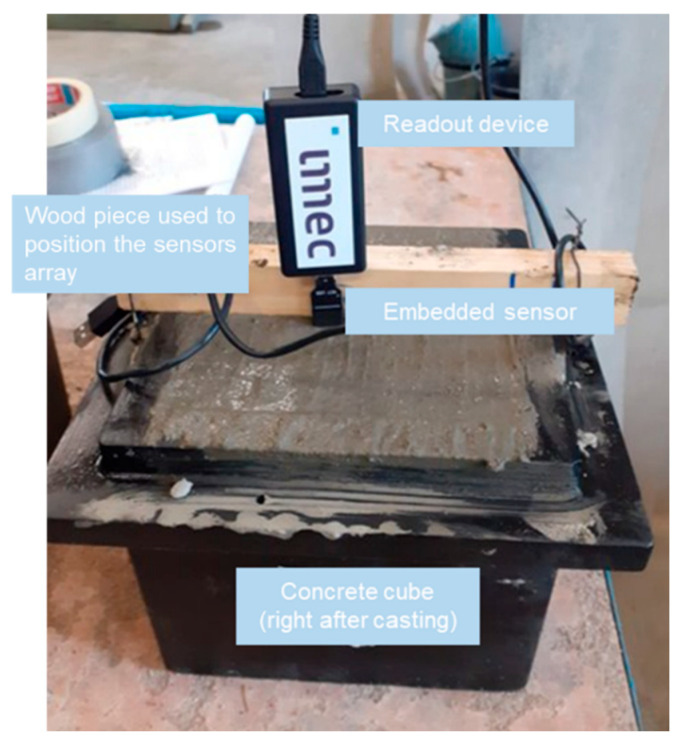
Experimental setup: sensor installation in concrete with readout for data acquisition.

Figure 3 shows the experimental setup. The ER sensor was embedded in the concrete cubes right after pouring and connected to a readout unit that would enable the communication of the sensor with the data collection unit. To ensure careful placing, the sensor was attached to a piece of wood fixed to the top of the mold. The sensor operated at a frequency of 3 kHz. At this frequency, the phase angle was 0, and, therefore, the impedance measurement of the ER could be used as a measure of resistivity of the concrete.

## 3. Results and Discussion

### 3.1. Sensitivity of the ER Sensors to Curing and Drying

In this section, we discuss the combined effects of curing and drying on the resistivity profiles obtained with the sensors. The results will be presented for each of the curing rooms described in Table 2 (considering Concrete A, without SAPs). To further highlight the effects of the curing conditions over the different levels, the variation rate of the resistivity (determined by the first derivative of resistivity over time) was also used. Once the effects of the curing conditions on the resistivity profiles were understood, it was possible to isolate one sensor level where the effects of drying (due to the difference in humidity between the curing environment and the concrete) were found to be minimal. This allows for further discussion on how the sensors could also be used to see different aspects of the hydration process of the cement under different curing conditions.

#### 3.1.1. Room 20/90—Reference Curing Room, Limited Effect of Drying

**Figure 4:**
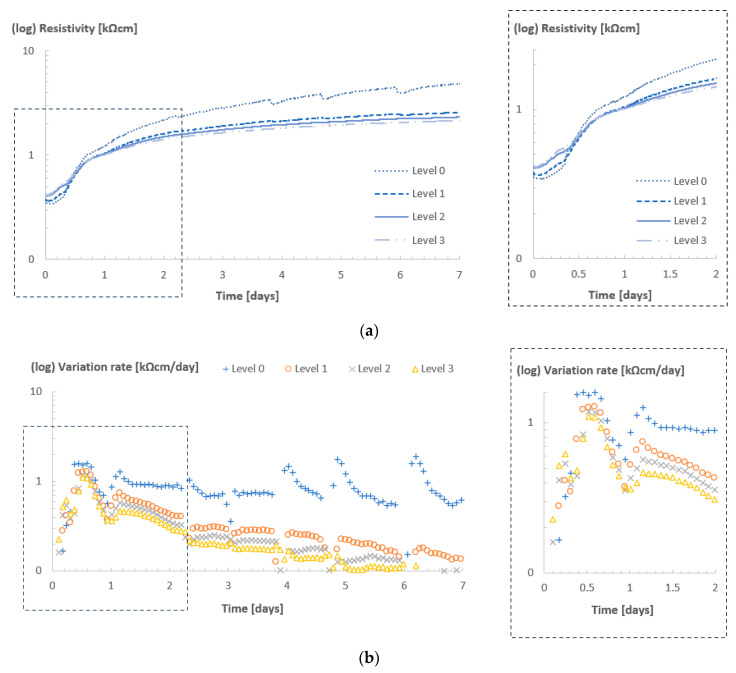
Resistivity (**a**) and variation rate (**b**) profiles for Concrete A in room 20/90.

Figure 4a shows the resistivity profile for the different sensor levels over time for Concrete A, in the ambient condition of room 20/90, and Figure 4b depicts the variation rate of the resistivity. 

In this room, due to higher ambient RH, evaporation/drying was expected to be minimal, especially in the innermost layers of the concrete cube. Therefore, changes in resistivity in the innermost levels were expected to be almost exclusively related to the ongoing hydration process of the cement. 

The resistivity values observed in all four levels were similar up to the first 24 h of curing. After that, with the cubes already demolded and with a higher exposure to environmental conditions, the outermost level presented considerably higher values of resistivity, which showcased the influence of drying on the resistivity measurement of the first sensor level (which was only 1–5 mm below the concrete surface). Even at a high relative humidity (in this case, above 90%), there was still an RH gradient between the air in the environment and the concrete specimen, which enabled drying to occur to some extent, especially near the surface.

In contrast, for all deeper levels the resistivity values were comparable, with a maximum difference below 0.5 kΩcm between levels 1 and 3, which could be considered as a strong indication that no severe drying was occurring at these levels and the evolution of resistivity was linked almost exclusively to the progressive cement hydration and the changes in the microstructure of the matrix during hardening. It was assumed that the concrete curing was developing in a uniform concrete cover depth covered by sensor levels 1, 2 and 3 (5–42 mm deep). 

The variation rate depicted in Figure 4b confirms the assumptions above. Overall, the rate at which resistivity changed for all levels indicated an increase during the initial hours (when the material was transitioning from a fluid to a solid state) and tended to stabilize in time, as the hydration process also tended to stabilize. Additionally, the older the concrete became, the influence of the drying decreased (since the humidity levels inside the concrete also tended to reach a lower and stable level with time, given the constant climate of the room). After one day, with the cubes demolded, the variation rates for all levels tended to decrease, but was always higher for level 0. In contrast, the variation rate for all other levels consistently decreased over time, which indicated that during the monitoring time of seven days, the external factors did not influence curing below the first 5 mm of the concrete covering.

#### 3.1.2. Room 20/60—Representative of Some Common Applications and Increased Influence from Drying

**Figure 5:**
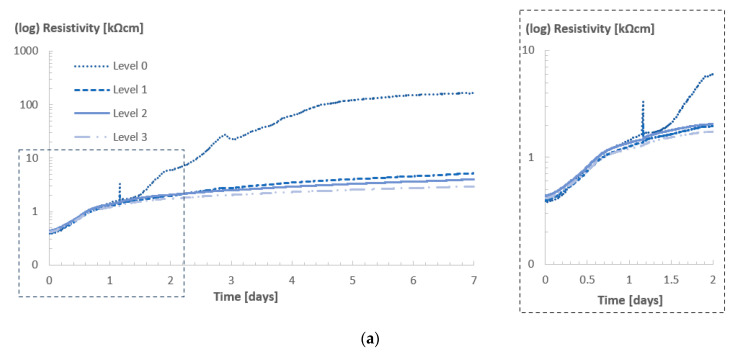

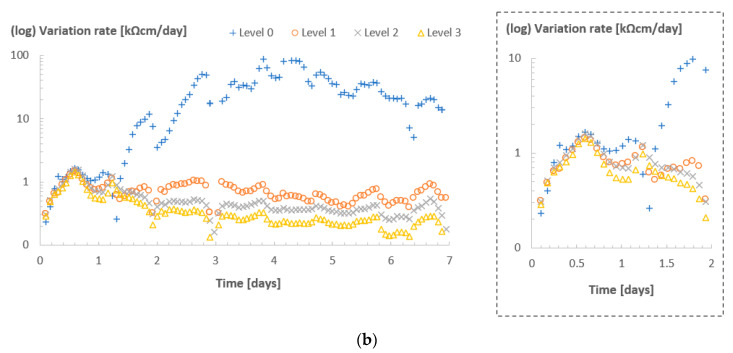
Resistivity (**a**) and variation rate (**b**) profiles for Concrete A in room 20/60. The “spike” observed in part (**a**) might be related to the disturbance on the specimen/sensor caused by transportation and demolding.

Figure 5a shows the resistivity values of Concrete A in room 20/60, and Figure 5b depicts the rate of variation of resistivity. 

In this room, due to lower ambient RH in comparison with room 20/90, evaporation/drying was expected to be higher, especially near the surface but not limited to it, as in time, the drying front could also reach (some of) the innermost levels, because the fresh concrete had higher internal RH (100%) in the beginning as compared with the ambient condition of room 20/60 (RH of 60%). 

During the first day of curing, the resistivity values for all levels were very similar, as well as the variation rate of the resistivity. Once the cubes were demolded and exposure to the curing environment increased, the resistivity values recorded at level 0 became significantly higher in comparison with the values registered in the other levels. One day after casting, the resistivity values near the surface of the concrete, i.e., level 0, were already seven to eight times higher than those in the inner levels, and from then onwards, the resistivity of level 0 grew exponentially until it reached a plateau. At day 7, the resistivity values of level 0 reached ca. 50 times the innermost level 3. In contrast, the difference between levels 1 and 3, and between levels 2 and 3, were remarkably lower.

**Figure 6:**
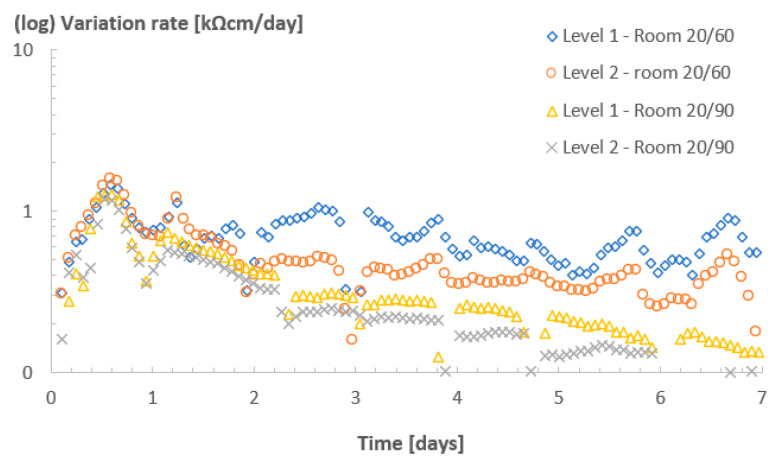
Comparison between variation rate curves for level 1 and 2 in rooms 20/60 and 20/90.

The variation rate for level 0 reached a maximum around 3 days after the casting and started to decrease after that. Additionally, for level 1, a noticeable difference in the variation rate was observed in comparison with level 3, indicating that the effects of drying were also affecting the resistivity values, thus the curing, at that level. In Figure 6, the variation rates of resistivity for levels 1 and 2 in both room 20/90 and 20/60 are depicted. By comparing the curves in the different rooms, it was clearly noticed that in room 20/90 there was a consistent decay in the rate of variation, especially after the setting of the concrete during the first day. In contrast, the rate of variation for levels 1 and 2 in room 20/60 were not only higher, but presented a less pronounced tendency of decaying from day 2 up to day 7 (especially for level 1), which could indicate that, indeed, drying was taking place up to level 2 for the concrete cube in room 20/60, given its lower ambient RH in comparison with room 20/90.

#### 3.1.3. Room 32/60—Representative of Applications during Summer Months, Higher Temperature, Faster Hydration, Increased Drying

**Figure 7:**
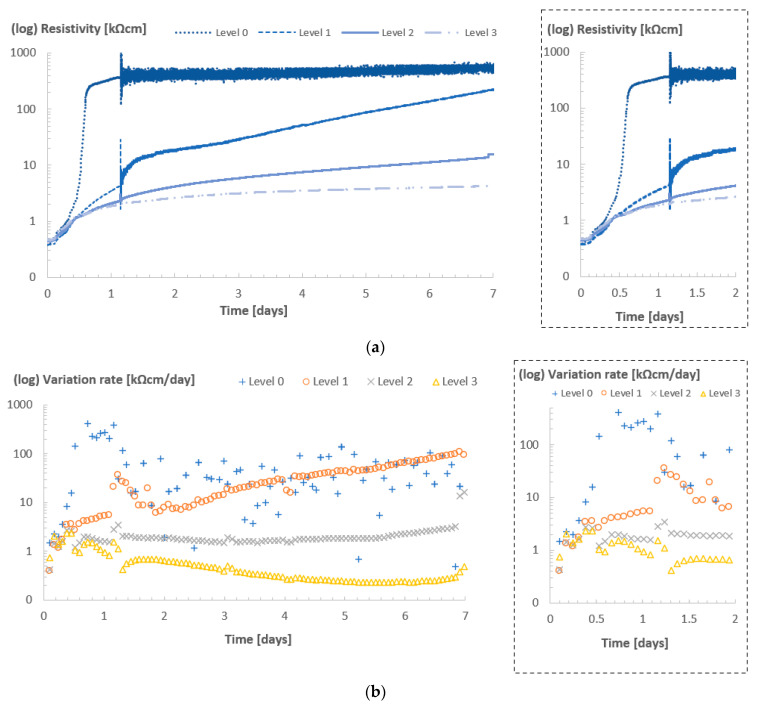
Resistivity (**a**) and variation rate (**b**) profiles for Concrete A in room 32/60. The “spike” observed in part (**a**) might be related to the disturbance on the specimen/sensor caused by transportation and demolding.

Figure 7 shows the resistivity values (a) and variation rate (b) for Concrete A in room 32/60. In this room, a different trend in the evolution of resistivity was expected due to the increased temperature compared with the other rooms. Higher temperatures accelerated the hydration reaction of cement, thus, accelerating water consumption and hardening.

The resistivity at level 0 grew exponentially from the very beginning of the monitoring period and reached values above 1000 kΩcm, which were 4, 80 and 300 times higher than those registered for levels 1, 2 and 3, respectively, at the same moment. Level 1 (5–11 mm) reached values of up to 350 kΩcm at 7 days, around 70 times higher than the value recorded in room 20/60 at the same age. A similar trend was also observed for level 2 (11–25 mm). The variation index also reflected such differences. The maximum rate of variation for level 0 was already achieved before 1 day, much earlier than in the other rooms. Additionally, for both levels 1 and 2, no decay in the rate of resistivity was observed. On the contrary, for these levels, an increasing trend was observed from day 2 up to day 7. 

Based on that, it is clear that the resistivity values and the curing process were significantly affected by the higher temperature, which accelerated the hydration reactions of the cement at the early ages. Furthermore, this acceleration not only meant a faster hardening and strength development, but also a faster water exchange, which, in time, added to the drying caused by the humidity gradient between the inside of the specimen and the atmosphere. Once again, the sensors captured such nuances.

#### 3.1.4. Highlighting the Effects of the Temperature on the Curing—A Comparison between Level 3 Curves

**Figure 8:**
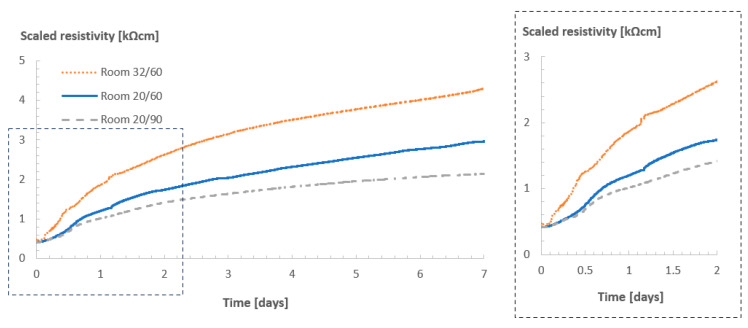
Resistivity values of sensors located at level 3 for Concrete A in different curing rooms.

With the analysis of the resistivity curves in all rooms, it was observed that in all cases the resistivity curves at the location of level 3 were minimally affected by the drying effect caused by the lower ambient RH of curing rooms 20/60 and 32/60, and the higher temperature of the latter (which also contributed to further evaporation). In a simplified way, it allowed the highlighting of only the effects of curing temperature on the hydration of the cement. Figure 8 shows the resistivity values at level 3 for concrete specimens in the different curing rooms.

The resistivity values from the concrete cube stored in room 32/60 were scaled to the temperature of 20 °C to allow an appropriate comparison with the previous experiments in room 20/60 and room 20/90. The temperature correction method followed Equation (1), as described in [30]. Here, *ρ_T_* and *ρ_Tref_* are the resistivity values of concrete (in kΩcm); *T* is the temperature inside the concrete (in °C); *T_ref_* is taken as 20 (in °C); *E_a-cond_* is the activation energy of conduction (here taken as 29.8 kJ/mol); and *R* is the universal gas constant (here taken as 8314 J/mol·k).

(1) $$\rho_{Tref}=\rho_{T}\;e^{\left[\frac{-E_{a-cond}}{R}\left(\frac{1}{T+273}-\frac{1}{T_{ref}+273}\right)\right]}$$

Under the same temperatures, the lower relative humidity affected curing at level 3, but to a very limited extent. In this regard, the curves from room 20/60 and room 20/90 were barely distinguishable up to the first 10–12 h of curing, and even after 7 days, the difference in resistivity value was lower than 1 kΩcm. In the case of the concrete in room 32/60, the scaled resistivity values were significantly higher. After 7 days of measurement, the difference between room 32/60 and room 20/60 reached ca. 5 kΩcm. As mentioned before, at high temperatures the rate of reaction for cement hydration increases, resulting in rapid densification of concrete [31]. Due to this rapid densification of concrete pores, the resistivity increases rapidly in the first 12 h. The rate of hydration reaction is highest in this time frame [32]. When varying ambient conditions, the resistivity values from ER sensor level 3 were significantly influenced by the cement hydration, whereas the ER sensor level 0 values were influenced by hydration as well as the ambient conditions. Therefore, ER sensor level 3 could be used to decouple the effect of drying from the hydration of cement.

**Figure 9:**
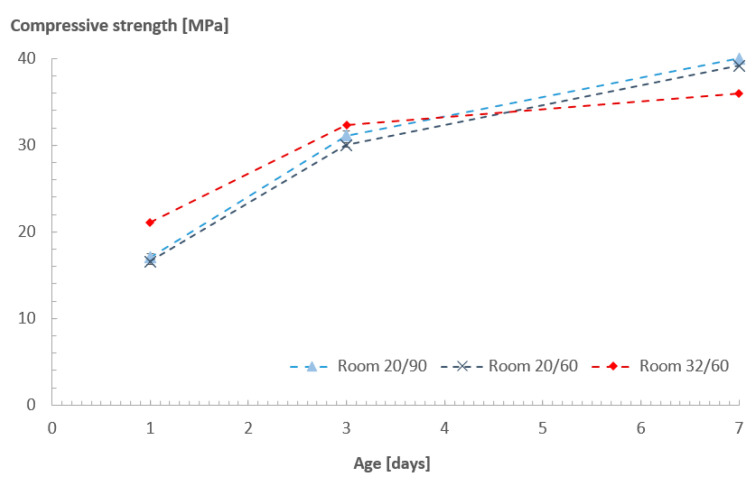
Compressive strength values of Concrete A.

Figure 9 shows the compressive strength measurement of the concrete in different ambient conditions at different ages using a standard testing procedure (standard NBN EN 12390-3:2009 [33]). The different effects of the curing conditions identified by the sensors also reflected differences observed in the strength development of the concrete mixtures. The cubes cured in rooms 20/90 and 20/60 had a similar strength development trend, with slightly higher strength values for the cubes stored in the room with higher humidity levels at 3 and 7 days (which can be directly related to the different resistivity patterns shown in Figure 8). As for the cubes stored in room 32/60, strength at day 1 was the highest of all, due to the accelerated hydration. However, the rate of strength development after that was lower in comparison with the other rooms. 

**Figure 10:**
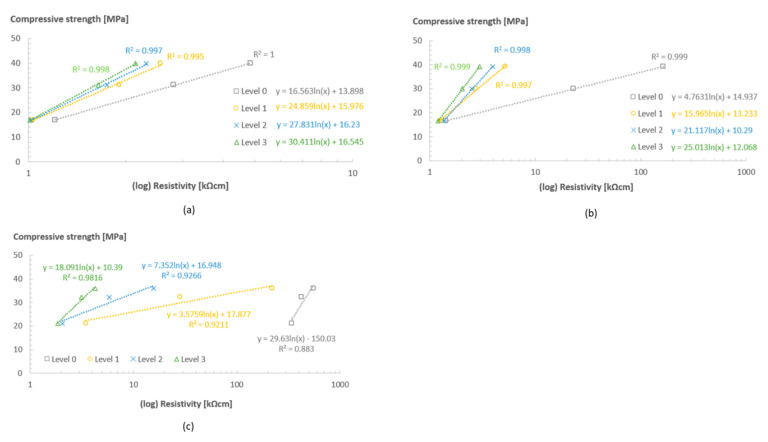
Compressive strength vs. (log) ER values at 1, 3 and 7 days for Concrete A in rooms 20/90 (**a**), 20/60 (**b**), and 32/60 (**c**).

The relationship between the compressive strength measured at 1, 3 and 7 days, and the ER values measured with the sensors, is depicted in Figure 10. In all levels, the data points are fitted with a logarithmic curve and the R^2^ is presented. In general, a high correlation between the strength and resistivity was found in all sensor levels in all rooms. For level 0 in room 32/60, there was a slight reduction in the R^2^ value when compared with all the other rooms and deeper sensor levels in the same room, which was associated with the faster strength development at the earlier ages (from 1 to 3 days) and stabilization right after that. Although there was still a very high correlation of the parameters in this sensor level, this could be an indication that for more extreme curing conditions, the use of data from locations near the surface of the structure could lead to lower correlations between the resistivity and other material properties. 

### 3.2. Effects of Internal Curing and Water Release from the Superabsorbent Polymers as Captured by the ER Sensor

**Figure 11:**
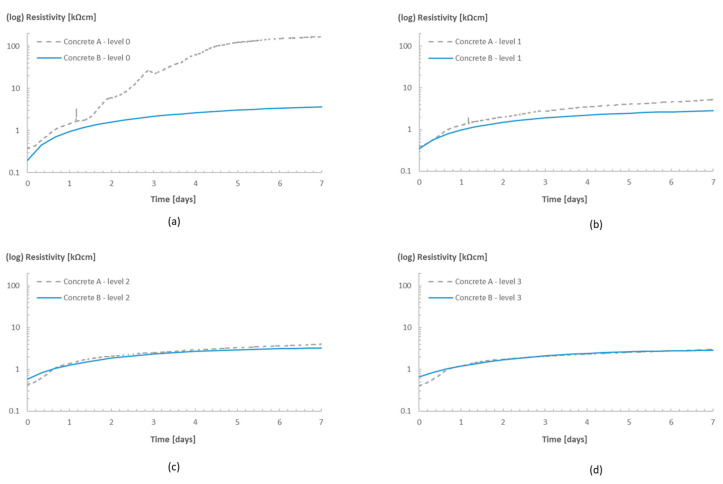
(**a**) Resistivity profiles for Concrete A and Concrete B in room 20/60: (**a**) level 0, (**b**) level 1, (**c**) level 2, and (**d**) level 3.

Figure 11 shows the resistivity profiles of the cubes containing superabsorbent polymers (SAPs), Concrete B, versus those of the reference mixture without the SAPs, Concrete A, also stored in room 20/60. 

The sensors were able to capture the internal curing effect of the SAPs in the concrete and provide some insights towards the water release of the polymers in the concrete. Disregarding some small differences during the first hours of monitoring (which could be related to the presence of air bubbles or even the swelling SAP particles in the vicinity of the sensors), the resistivity values of both concrete mixtures at levels 0 and 1 were comparable during the first 12–24 h of measurements. After that, however, the curves started to diverge, which was not so pronounced at levels 2 and 3. The reference concrete reached the higher resistivity values over time. In addition, the moment where the curves started to diverge also shifted to a later time for deeper levels. The explanation for that lies in the kinetics of water sorption and desorption in hardening cementitious materials. During the mixing of the concrete with SAPs, they are added to the dry materials in the form of a dry powder, which is uniformly distributed over the volume of the material. Once water is added to the mixture, the SAPs absorb the amount of water intended for internal curing and become gel-like water-filled reservoirs. Later, once the mixing water is consumed by the cement hydration, and the humidity levels of the hardening concrete start to drop, the water stored in the SAPs starts to progressively be released to maintain the high humidity levels. This water release might be associated with the reduction in the resistivity values observed in levels 0, 1, and 2. 

As the humidity in room 20/60 was lower than the humidity inside the fresh concrete cube, evaporation also played an important role in the water movement inside the concrete. Not only did the cement hydration consume water, but the humidity gradient also increased due to the water loss to the environment, which accelerated the drying process in the layers of concrete closer to the surface. The drying of the outer layers then forced the release of water from the SAPs prematurely, which is observed in Figure 11 (more pronounced for level 0), where the resistivity values for the internally cured concrete are lower than those of the reference concrete from the start, due to increase in the humidity values of the concrete near the sensor. The deeper the location of the sensor in the concrete, the longer it takes for the curves to diverge, representing the delay in the water release by the SAPs. For levels 2 and 3 (especially), no divergence was observed in the resistivity curves, which might indicate that no water was being released from the SAPs. 

## 4. Conclusions

The quality control of concrete during construction is critical for ensuring the long-term durability and service life of concrete infrastructure. Measuring fresh and early age concrete properties, as well as the efficiency of the curing process, can provide valuable information that can help to ensure the quality control of concrete. In this study, we presented a cost-effective PCB-based array of resistivity sensors that can measure concrete resistivity at different depths from the fresh state of concrete through early age development. 

The sensor was able to detect the effects of drying over different layers in the concrete depth, under different drying conditions, and to characterize the acceleration of the hydration at early ages when the concrete was subjected to higher temperatures. In addition, the sensors were able to distinguish the drying pattern of concrete mixtures with and without superabsorbent polymers used as curing agents, and provided insights allowing for the identification of the water release by superabsorbent polymers when used as internal curing agents. This might represent the possibility of using the sensors as a new testing method when investigating the efficiency of SAPs for use in concrete.

When correlating the strength development of the concrete under different curing conditions with the values of resistivity measured at different locations, it was observed that for curing conditions without severe drying or faster hydration, there was a strong correlation between compressive strength and resistivity, regardless of the location where the resistivity was measured. In contrast, when the concrete was subjected to higher curing temperatures and more severe drying conditions, although the correlation between compressive strength and resistivity was still high, it was also slightly lower for the resistivity values measured near the concrete surface in comparison with the rooms with less severe curing conditions. 

In summary, the solution presented in this exploratory study has the potential to enable the implementation of a network of sensors throughout the structure, providing more representative values and better estimates of strength development and curing conditions, ultimately ensuring the long-term durability of concrete infrastructure.

## Figures and Tables

**Table 1:** Composition of the concrete mixtures.

Materials [kg/m^3^]	Concrete A	Concrete B
Cement (type CEM I 52.5 N)	300	300
River sand 0/4 mm	665	665
Gravel 2/8 mm	575	575
Gravel 8/16 mm	675	675
Superabsorbent polymers	0	1.14
Water (mixing)	170	170
Water (internal curing)	0	23.94

**Table 2:** Details of the ambient conditions in different concrete curing rooms.

Room	Temperature [°C]	Relative Humidity [%]	Comments
Room 20/90	20 ± 2	≥90	Reference curing room, high humidity level, slow drying, optimal curing in accordance to the standard NBN EN 12390-2:2019 [29].
Room 20/60	20 ± 2	60 ± 5	Representative of some common applications.
Room 32/60	32 ± 2	60 ± 5	Representative of applications during summer months, higher temperature, faster hydration, increased drying.

**Table 3:** Details of the electrode design.

Parameter	Value
Electrode area	2 mm × 4 mm
Electrode spacing level 0	4 mm
Electrode spacing level 1	6 mm
Electrode spacing level 2	14 mm
Electrode spacing level 3	17 mm
Length of sensor embedded in concrete cover depth	50 mm

## Data Availability

The data presented in this study are available on request from the corresponding author. The data are not publicly available due to intellectual property rights involving the industrial partners of the project.
